# Lipid Matters: How Herbicidal Ionic Liquids Engage with Membranes

**DOI:** 10.1021/acs.jpcb.5c07127

**Published:** 2026-01-21

**Authors:** Aleksandra Bagińska, Anna Syguda, Katarzyna Dopierała

**Affiliations:** Institute of Chemical Technology and Engineering, 431508 Poznan University of Technology, Berdychowo 4, 60-965 Poznań, Poland

## Abstract

This study explores the influence of alkyl chain length and concentration of herbicidal ionic liquids based on (4-chloro-2-methylphenoxy)­acetic acid (MCPA) on the structural and mechanical properties of model lipid monolayers composed of phospholipids: 1,2-dimyristoyl-*sn*-glycero-3-phosphoethanolamine (DMPE), 1,2-dipalmitoyl-*sn*-glycero-3-phosphocholine (DPPC), and sterols (cholesterol, ergosterol). Using the Langmuir monolayer technique, we demonstrate that the effects of [Cn]­[MCPA] on lipid films strongly depend on both the alkyl chain length and the herbicide concentration. Among the studied compounds, ionic liquids with dodecyl and tetradecyl chains exhibited the most pronounced fluidizing and expanding effects. Dose-dependent experiments revealed that increasing [C12]­[MCPA] concentrations enhance monolayer expansion and fluidization, with magnitudes varying across lipid types. Penetration experiments simulating dynamic herbicide-membrane interactions indicate a substantial ability of [C12]­[MCPA] to insert into condensed DMPE and ergosterol monolayers, while insertion into cholesterol and DPPC films is limited. The diverse responses of lipid membranes to [C12]­[MCPA] suggest the potential to modulate herbicide selectivity toward target and nontarget cell membranes, thereby improving both efficacy and environmental safety. Overall, our findings elucidate key structure–activity relationships governing membrane perturbation by herbicidal ionic liquids and validate Langmuir monolayer studies as an efficient in vitro approach for predicting the biological activity of membrane perturbants. This work provides molecular-level insights that can guide the rational design of selective and safe herbicidal agents.

## Graphical abstract

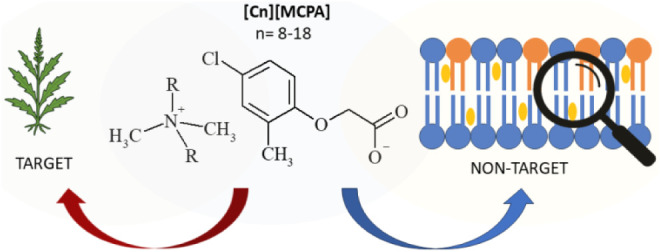

## Introduction

In modern agriculture, the escalating global demand for food renders the use of plant protection products inevitable. Desired and undesired plants compete for light, water, and nutrients, necessitating the use of selective herbicides, such as phenoxy acids. Among these, (4–chloro–2–methylphenoxy)­acetic acid (MCPA) is widely used due to its high phytotoxicity toward most dicotyledonous plant species, including cornflower, while sparing monocotyledonous plants, such as cereals. Functioning as a synthetic auxin, MCPA mimics natural growth hormones, perturbing the hormonal balance, protein synthesis, and plant developmental processes. Its systemic action enables uptake through weed foliage, leading to tissue deformation, growth inhibition, and ultimately plant death, with peak efficacy observed at the 2–6 leaf developmental stage.^1–3^

Commercial MCPA formulations are available as sodium–potassium salts (e.g., Chwastox Extra 300 SL), protonated ammonium salts, such as dimethylammonium salt (e.g., Dicoherb 750 SL), and esters, such as 2–ethylhexyl alcohol ester (e.g., Chwastox AS 600 EC).^4,5^ Ester derivatives typically offer superior herbicidal performance due to enhanced penetration through the leaf cuticle; however, their high volatility poses a risk to neighboring dicotyledonous crops, such as grapevines, and may be harmful to humans conducting spraying. In contrast, salt forms (either protonated ammonium or sodium–potassium) adsorb more slowly but produce a milder effect.^6^

While MCPA salts primarily suppress plant growth, the impact of their ionic form on other living organisms is more complex. Beyond plants, these compounds can affect soil microorganisms and aquatic biota, with toxicity profiles and bioavailability modulated by the accompanying cation.^7–9^

Despite MCPA’s well-established selectivity toward dicotyledonous plants, important questions remain concerning resistance mechanisms and interactions with other active agents.^1,10,11^ Aquatic toxicity of MCPA and potential accumulation in water systems remain poorly characterized,^2^ and the impact of MCPA on soil microorganisms—critical to soil health and nutrient cycling—warrants deeper investigation,^11,12^ especially concerning disruption of plant–microbe symbioses.^13,14^

Herbicidal ionic liquids (HILs) have emerged as an innovative approach in sustainable agriculture, aiming to reduce the environmental footprint of herbicides by improving target selectivity and minimizing volatility.^15^ These organic salts with melting points below 100 °C contain ions that exert phytotoxic effects on undesirable plant species.^16^ As an alternative to traditional formulations, MCPA-based HILs with dialkyldimethylammonium cations exhibit increased wettability of weed leaves, allowing for lower application doses and minimizing the risk of water and soil contamination.^15,17^ However, ecotoxicological uncertainties persist, particularly concerning nontarget organisms and ecosystem balance. In soils, HILs containing the MCPA anion may influence not only weed populations but also microorganisms that are essential for plant health and soil stability. These microorganisms, which participate in nutrient cycling and organic matter degradation, could be adversely affected by MCPA interactions with their cell membranes, potentially disrupting plant–microbe symbiosis and leading to unforeseen consequences for soil health. Analogous concerns arise for aquatic ecosystems, where contamination could pose chronic risks, yet long-term toxicity and bioaccumulation data remain scarce.

Toxicological profiles of HILs are intricately linked to their interactions with cell membranes—the primary biological barrier to xenobiotics. Lipid bilayers are dynamic, highly selective environments, now recognized to play a far more active role than previously thought.^18^ As shown in Table 1, the lipid composition of cell membranes varies significantly between species, and even small changes in distribution may produce profound effects in biological events.^19^

**Table 1:** Typical Features and Distribution of Phosphatidylcholine (PC), Phosphatidylethanolamine (PE), Cholesterol (CHOL), and Ergosterol among Living Organisms

lipid type	structural and functional features	distribution	refs
PC	• Zwitterionic, the choline bears a positive charge, while phosphate contributes a negative charge; neutral charge overall	• Major component of the outer leaflet of animal cell membranes (up to 50% of phospholipids)	^20–23^
	• Cylindrical geometry favors bilayer formation	• Common in most eukaryotic organisms	
		• Rare in bacterial membranes	
		• Makes up to 80% of pulmonary surfactant	
PE	• Headgroup with ethanolamine, typically cone-shaped due to small size; zwitterionic at neutral pH;	• Enriched in inner leaflet of animal and plant cell membranes; abundant in mitochondrial membranes (up to 35%)	^24–26^
	• Neutral or slightly negative charge	• Present in Gram-negative bacterial and plant membranes	
	• Tends to adapt hexagonal arrangements		
CHOL	• Built from a rigid four-ring structure with a small hydroxyl headgroup; uncharged	• Main sterol in animal cell membranes	^27–29^
		• Highly abundant in animal tissues (e.g., brain, nerves)	
	• Regulates membrane fluidity, rigidity, and permeability	• Absent in plant and bacterial membranes	
ERG	• Structurally similar to cholesterol but contains two additional double bonds and a methyl group	• Dominant sterol in fungal membranes (e.g., yeast)	^28^,^30–32^
		• Total amount depends on species, age, and growth conditions	
	• Influences membrane curvature and rigidity	• Not found in animal cell membranes	
		• Absent or minor component of higher plant membranes	

*In vitro* methodologies are increasingly employed to evaluate the effects of biologically active compounds on both target and nontarget organisms at the membrane level. These approaches provide early indicators of oxidative stress and other toxic responses.^18–34^ While *in vitro* studies cannot fully capture the complexity of living organisms, potentially leading to discrepancies when extrapolating results to *in vivo* context, they offer significant ethical and practical advantages by reducing reliance on human or animal testing.^35–37^ Artificial cell membranes, in particular, enable detailed mechanistic investigations on the cellular and molecular scales, offering a cost-effective approach.

This work presents a molecular-scale study of how the chemical structure of herbicidal ionic liquids containing the MCPA anion modulates their interactions with lipid monolayers. We selected four representative lipids, which constitute the principal components of cell membranes in nontarget organisms (Table 1). We hypothesize that the biological impact of MCPA salts can be correlated to the lipid composition of the affected membrane. Furthermore, we propose that the monolayer approach provides a valuable screening tool for the preliminary evaluation and prediction of the herbicide effects on nontarget organisms. This work contributes to a deeper understanding of the off-target effects of MCPA-based herbicides, including their dose-dependent behavior and molecular mechanisms of action.

## Materials and Methods

### Materials

The herbicidal ionic liquids used in this study—dialkyldimethylammonium (4-chloro-2-methylphenoxy)­acetates ([Cn]­[MCPA])—were synthesized following the procedure described earlier.^17^ The purity of the cationic surfactant was determined by two-phase titration (EN ISO 2871-1.2:2010). The remaining amount, up to 100%, was verified as water by coulometric Karl Fischer titration, as described in another study.^38^ The complete list of synthesized compounds, along with their abbreviations and corresponding cation structures, is provided in Table 2.

**Table 2:**
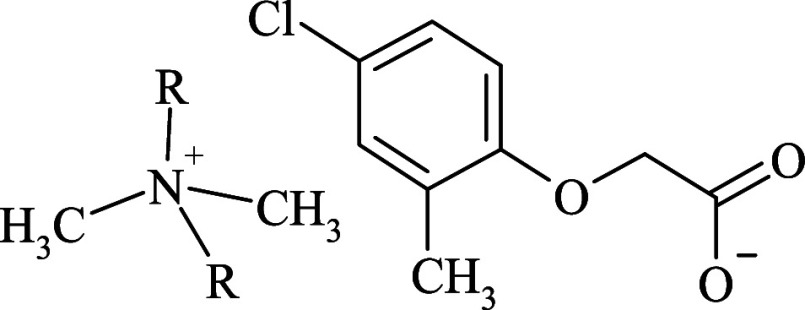
Dialkyldimethylammonium (4-chloro-2-methylphenoxy)­acetates used in the study

R	abbreviation of HIL	name of the cation	purity [%]
C_8_H_17_	[C8][MCPA]	dimethydioctylammonium	98.0
C_10_H_21_	[C10][MCPA]	didecyldimethylammonium	99.5
C_12_H_25_	[C12][MCPA]	didodecyldimethylammonium	99.0
C_14_H_29_	[C14][MCPA]	dimethylditetradecylammonium	98.5
C_16_H_33_	[C16][MCPA]	dihexyldimethylammonium	96.5
C_18_H_37_	[C18][MCPA]	dimethydioctadecylammonium	95.0

The lipids used in monolayer experiments were 1,2-dipalmitoyl-*sn*-glycero-3-phosphocholine (DPPC, semisynthetic, ≥99%, Sigma-Aldrich), 1,2-ditetradecanoyl-*sn*-glycero-3-phosphoethanolamine (DMPE, synthetic, ≥99%, Sigma-Aldrich), cholesterol (CHOL, ≥99%, Sigma-Aldrich), and ergosterol (ERG, 87.6%, Sigma-Aldrich).

## Methods

### Surface Pressure–Area Isotherms

All monolayer experiments were conducted using a Teflon Langmuir trough (KSV NIMA, Helsinki, Finland) with a total surface area of 273 cm^2^, thermostated at 21.0 ± 0.1 °C with a Julabo temperature control unit. Ultrapure water (resistivity of 18.2 MΩ·cm; ELGA Purelab) was employed as the subphase. Surface pressure (π) was measured with a platinum Wilhelmy plate connected to a precision balance and monitored by KSV NIMA software, providing a resolution of 4 μN/m and an accuracy of 0.1 mN/m.

Each lipid was dissolved in chloroform (UVASOL, Merck) to a final concentration of 1 mg/mL. The purity of the subphase was verified before each measurement; any surface impurities were removed by aspiration until the surface pressure at maximum compression was ≤0.20 mN/m. Lipid monolayers were formed by carefully spreading the solution onto the air–water interface using a microsyringe. The optimal spreading volume was determined experimentally to ensure that the initial surface pressure remained below 0.50 mN/m. After solvent evaporation (15 min), the films were compressed symmetrically by the barriers at a constant rate of 10 mm/min. During compression, the mean molecular area (*A*) was continuously recorded to obtain the surface pressure–area isotherm.

All measurements were replicated, yielding the reproducibility of the mean molecular area within ± 3 Å^2^/molec. Using these data, the compression modulus (*C*_s_^–1^) was determined according to the following equation:^39^

1 $${C_{s}}^{-1}=-A{\left(\frac{\Delta \pi }{\Delta A}\right)}_{T}$$

in which *A* represents the mean molecular area, π is the surface pressure, and *T* is the temperature. Based on the maximum values of the compression modulus, the physical states of monolayers can be distinguished as gaseous (G) for *C*_s_^–1^_max_ < 12.5 mN/m, liquid-expanded (LE) for 12.5 mN/m < *C*_s_^–1^_max_ < 50 mN/m, liquid (L) or L/LE coexisting phase for 50 mN/m< *C*_s_^–1^_max_ < 100 mN/m, liquid-condensed (LC) for 100 mN/m< *C*_s_^–1^_max_ < 250 mN/m, and solid (S) for *C*_s_^–1^_max_ > 250 mN/m.

From the recorded surface pressure–area isotherms, the collapse pressure (π_coll._) was determined as the surface pressure at which the two-dimensional film lost stability and three-dimensional structures began to form at the interface. Data acquisition, analysis, and visualization were performed using Origin 8.5.1 software (OriginLab, USA).

The π–A isotherms for lipid–herbicide systems were obtained under the same experimental conditions, with variations arising solely from differences in the subphase composition. Experiments involving [Cn]­[MCPA] were conducted by introducing an aqueous solution of the herbicide (*c* = 1.72 mM/L) into the clean subphase (250 mL) prior to lipid spreading. The volume of the added herbicide solution was adjusted to yield final concentrations in the subphase, ranging from 0.07 to 0.69 μmol/L.

### Penetration Experiments

Penetration experiments were performed following a protocol analogous to that used for recording the π–A isotherms, with the key difference that the lipids were spread first and compressed to the target surface pressure of π = 30 mN/m. Upon reaching this target, an aqueous solution of [C12]­[MCPA] (*c* = 1.72 mM) was injected into the subphase using a microsyringe. Two distinct concentrations of [C12]­[MCPA] were tested to assess the concentration-dependent effects. Throughout the experiment, the surface pressure was held constant, and the response of the lipid monolayer was monitored by recording changes in the mean molecular area as a function of time. The results were expressed as *A*/*A*_0_(t) curves, where *A* represents the mean molecular area at time *t* and *A*_0_ denotes the mean molecular area at *t* = 0, corresponding to the moment of herbicide injection.

## Results and Discussion

### Effect of Alkyl Chain Length

Figure 1 presents the π–A isotherms of lipid monolayers in the presence of 0.41 μmol/mL [Cn]­[MCPA] salts with varying alkyl chain lengths alongside the corresponding isotherms of pure lipids for comparison. The data indicate a noticeable influence of the salts on monolayer behavior, which can be evaluated by examining shifts in the collapse point, lateral displacement along the mean molecular area axis, and changes in the slope of the isotherms.

**Figure 1:**
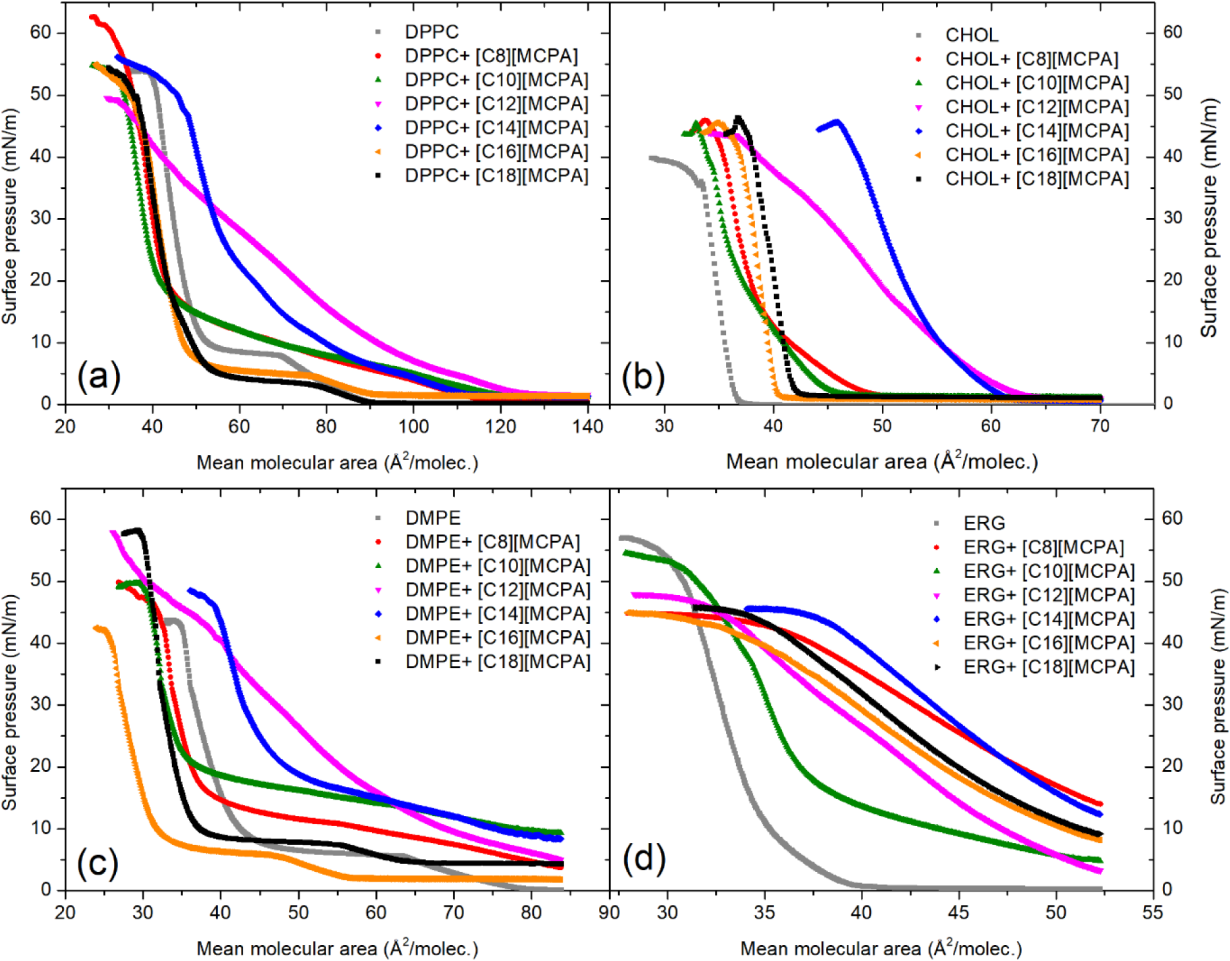
Π–A isotherms of DPPC (a), CHOL (b), DMPE (c), and ERG (d) monolayers in the presence of 0.41 μmol/L of [Cn]­[MCPA] salts in the aqueous subphase recorded at 21 °C at a compression rate of 10 mm/min. The amount of lipid was kept constant in each group of experiments to keep the same lipid: herbicide ratio.

The DPPC monolayer (Figure 1a) shows a characteristic plateau between 51–70Å^2^/molec., a sharp rise at 50Å^2^/molec., and collapses at ∼55 mN/m. The presence of [C12]­[MCPA] and [C14]­[MCPA] induces a notable shift of the isotherm toward larger mean molecular areas, which can be attributed to the adsorption and incorporation of these salts into the monolayer. Conversely, salts with longer alkyl chains produce an opposite effect: the plateau remains largely unchanged, suggesting the weak affinity of these salts for the DPPC monolayer and minimal disruption of membrane organization. Furthermore, the collapse pressures (π_coll._) in the presence of [C16]­[MCPA] and [C18]­[MCPA] remain comparable to those of pure DPPC, indicating that the mechanical stability of the monolayer is largely preserved.

In the case of the cholesterol monolayer (Figure 1b), all [Cn]­[MCPA] salts have strong affinity for the film, inducing a shift of the curves toward larger molecular areas. Similar to observations for DPPC, the most evident effects are observed for [C12]­[MCPA] and [C14]­[MCPA]. Notably, all salts increase π_coll._ by approximately 10 mN/m; however, significant changes in the slope of the isotherms occur only for *n* ≤ 14, indicating a chain-length-dependent modulation of monolayer packing.

For the DMPE monolayer (Figure 1c), the influence of [Cn]­[MCPA] is evident even before compression, as indicated by a rise of the initial surface pressure immediately after spreading the lipid. This effect is the most significant for *n* = 10 and 14 and is absent in the DPPC system. It means the interactions between DMPE and [Cn]­[MCPA] are visible even before mechanical compression. This behavior likely arises from the smaller headgroup of the DMPE molecule, which facilitates more efficient adsorption of soluble salts at the interface. A similar phenomenon was previously observed for 1-alkyl-1-methylpiperidinium bromides interacting with model fungal membranes.^37^ In contrast, DMPE monolayers in the presence of [C16]­[MCPA] and [C18]­[MCPA] exhibit a visible LE-LC plateau analogous to the DPPC film, indicating only minor perturbations of molecular organization. Most DMPE monolayers with [Cn]­[MCPA] collapse at higher surface pressure than pure DMPE, reflecting enhanced monolayer stability, except [C16]­[MCPA], which does not alter π_coll._ of DMPE.

For the ERG monolayer, an increase in the initial surface pressure is observed in the presence of all [Cn]­[MCPA] salts, similar to the DMPE film but unlike CHOL. All HILs induce monolayer expansion, as evident from the π–A isotherms in Figure 1d, with the curves shifted toward larger molecular areas. However, unlike CHOL, ERG monolayers are destabilized by the salts, as indicated by lower π_coll._ values compared to pure ERG. The greatest expansion of ERG monolayers occurs with [C14]­[MCPA], while the effect of [C12]­[MCPA] is less pronounced than that for CHOL.

In monolayer studies, a surface pressure of π = 30–35 mN/m is often used to approximate the lateral pressures found in biological membranes, where packing density and domain formation resemble those in natural bilayers. Accordingly, in Figure 2, we analyzed the changes in mean molecular areas at a fixed π = 30 mN/m (ΔA_30_) induced by [Cn]­[MCPA] with varied alkyl chain lengths. This analysis enables a direct comparison of the effects of each HIL on different lipid monolayers. Positive ΔA_30_ values indicated monolayer expansion caused by the salts, whereas negative ΔA_30_ values reflect a condensing effect on the lipid film.

**Figure 2:**
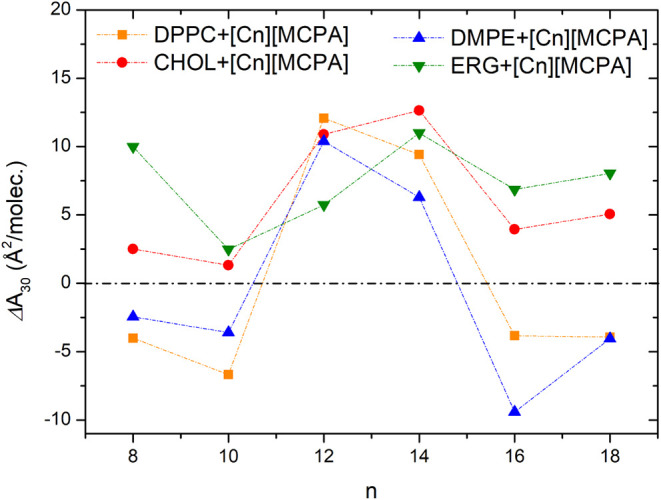
Mean molecular area change at *π* = 30 mN/m (ΔA_30_) determined from the π–A isotherms shown in Figure 1 as the difference between the molecular area of pure lipid and the mean molecular area of the same lipid in the presence of [Cn]­[MCPA]. The lines are shown only as guides for the eye and do not represent a physical model.

From the data presented in Figure 2, it is evident that the alkyl chain length in [Cn]­[MCPA] distinctly influences the mean molecular area of the individual lipids. Even when considering a 3 Å^2^ uncertainty in monolayer experiments, HILs with *n* = 12 and 14 display behavior that clearly differs from salts with *n* = 8, 10, 16, and 18. The dodecyl and tetradecyl homologues induce a strong expansion of phospholipid monolayers, whereas the presence of shorter (*n* = 8, 10) or longer (*n* = 16, 18) chains leads to condensation of DPPC and DMPE films. The pronounced expansion of the phospholipid monolayers observed for the dodecyl and tetradecyl derivatives suggests that these alkyl chain lengths may facilitate insertion into cellular membranes enriched in these lipid components, such as the outer leaflet of the animal plasma membrane and bacterial membranes.

In contrast, no condensing effect is observed for sterols. Both CHOL and ERG exhibit a consistent expansion of the molecular area in the presence of all of the [Cn]­[MCPA] salts studied. This finding implies that sterol-rich domains, often associated with lipid rafts, may be relatively resistant to HIL-induced perturbations, thereby potentially preserving the microdomain organization. Furthermore, the ΔA_30_ values for CHOL and ERG shown in Figure 2 remain, in most cases, within experimental error. This observation indicates that structural differences between these two sterols do not significantly affect their interactions with HILs. Overall, the contrasting responses of phospholipids and sterols to [Cn]­[MCPA] can be attributed to their fundamental structural features: the rigid, four-ring core of sterols versus the linear, flexible hydrocarbon chains of phospholipids. As illustrated in Figure 1, the incorporation of [Cn]­[MCPA] salts generally alters the slope of the π–A isotherms compared with that of pure lipid monolayers, suggesting that salt incorporation modifies monolayer fluidity.

To further elucidate this effect, we calculated the compression modulus using eq 1 and plotted the results against the mean molecular area in Figure 3. The intrinsic properties of the lipids strongly influence the membrane fluidity, as evidenced by the maximum *C*_s_^–1^ values for DPPC, DMPE, CHOL, and ERG. DPPC forms a more fluid monolayer compared with sterols and DMPE, with a maximum *C*_s_^–1^ of 225 mN/m. In contrast, DMPE generates a more tightly packed film due to a less bulky headgroup. This observation aligns with previously reported findings.^40–43^ Notably, high *C*_s_^–1^ corresponds to reduced lateral elasticity, reflecting the dense packing of the lipids in the monolayer.

**Figure 3:**
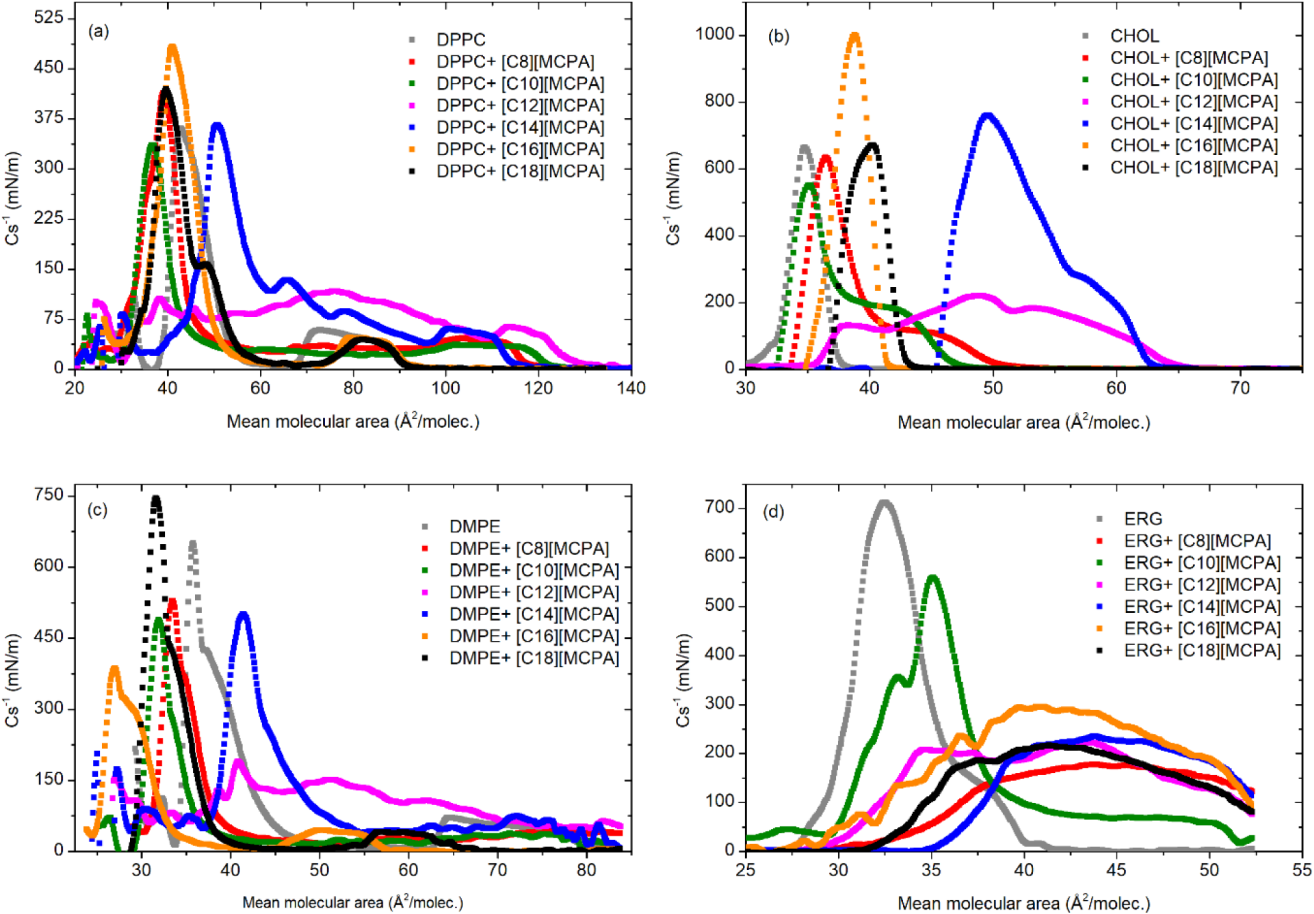
Compression modulus curves determined from eq 1 using the π–A isotherm data for DPPC (a), CHOL (b), DMPE (c), and ERG (d) monolayers.

The compression modulus of the DPPC monolayer is most strongly reduced by [C12]­[MCPA], while that of CHOL is most strongly reduced by [C10]­[MCPA], DMPE is most strongly reduced by [C16]­[MCPA], and ERG is most strongly reduced by [C8]­[MCPA]. These changes are most noticeable for DPPC and ERG, indicating the greatest fluidizing effect on these lipid membranes. Interestingly, for the cholesterol monolayer, exposure to [C8]­[MCPA] led to a marked increase in compression modulus, suggesting an enhanced rigidity—a trend likewise observed for DPPC under similar conditions.

In the case of DMPE, the monolayer becomes more fluid in the presence of nearly all salts except for [C18]­[MCPA], which preserves a more rigid packing. In contrast, ERG exhibits a consistent and substantial decrease in *C*_s_^–1^ across all HILs tested (Figure 3d), indicating a strong tendency toward increased fluidity. For CHOL, notable changes in the isotherm slope are apparent only for homologues with *n* ≤ 14. In particular, the compression modulus plot in Figure 3b reveals a significant reduction in *C*_s_^–1^ with [C12]­[MCPA], corresponding to a phase transition from a solid to a liquid-condensed state. Such alterations in membrane fluidity are crucial for cell viability as they directly influence essential physiological processes. Membrane fluidity governs not only cellular integrity but also fundamental biological functions, such as nutrient transport, signaling, and the maintenance of homeostasis.^24,44^ Modulating membrane fluidity has recently emerged as a promising strategy in modern therapies, including anticancer applications.^45,46^ In this context, the distinct and in some cases opposite effects of [Cn]­[MCPA] salts on lipid monolayer fluidity may serve as valuable predictors of their biological activity within specific cellular environments. For herbicidal compounds, such physicochemical insights could provide an early indication of molecular mobility and a preliminary marker of potential toxicity toward non-target organisms.

It must be acknowledged that natural biological membranes are asymmetric bilayers containing a complex mixture of lipids and proteins, which imposes certain limitations on the monolayer approach.^22^ Nonetheless, analyzing individual lipid components in controlled monolayers enables a precise assessment of the affinity of [Cn]­[MCPA] toward distinct membrane types of lipid domains enriched in specific phospholipids or sterols. These experiments also help identify membrane regions that might be particularly susceptible to perturbation by a given compound.

Based on data presented in Figure 1–3, it can be concluded that [Cn]­[MCPA] salts exhibit varying affinity to the tested lipids, with several homologues showing the ability to incorporate into and expand monolayers—especially those containing sterols. Among the derivatives studied, [C12]­[MCPA] demonstrates the strongest potential to fluidize all examined monolayers, highlighting its pronounced interaction with diverse lipid environments.

Our findings are consistent with numerous reports highlighting the exceptional biological activity of organic salts bearing dodecyl and tetradecyl alkyl chains. For instance, cationic gemini surfactants with chain lengths of *n* = 10–14 have been shown to alter erythrocyte membrane fluidity.^47^ Piperidinium-based ionic liquids containing the mandelate ion exhibit the highest antibacterial activity against Gram-positive bacteria when their alkyl chain is C12.^48^ Likewise, choline-derived ionic liquids show maximal antibacterial efficiency for the C12 homologue.^49^ A comparable trend was reported for tetraalkylammonium salts derived from 4-*N*-methylmorpholine, where derivatives with dodecyl to hexadecyl chains demonstrated the strongest bactericidal effects.^50^ Excellent biological efficiency results were also reported for a dichlorprop-based herbicidal ionic liquid with a dodecyltrimethylammonium cation.^51^ Moreover, variations in cytotoxicity linked to alkyl chain length have been recorded for homologous series of imidazolium salts, with maximum membrane disruption and cytotoxicity observed for chains containing 15 carbon atoms in the alkyl chain.^52^

### Effect of [C12]­[MCPA] Concentration on Monolayer Behavior

Interactions between herbicides and both target and nontarget organisms are generally dose-dependent. To gain deeper insight into the impact of the investigated HILs on membrane lipids, we examined the response of the lipid monolayer to varying concentrations of [C12]­[MCPA] introduced into the subphase. This particular salt was selected based on the most promising results across all [Cn]­[MCPA] salts in this study.

The concentrations of the herbicide investigated in this study were below the expected critical micelle concentration (CMC) value. The CMC for [C10]­[MCPA] has been determined previously^15^ and is equal to 0.251 mM, with a corresponding surface tension at CMC γ_CMC_ = 26.2 mN/m. For homologous series of surfactants, CMC values typically span no more than two or 3 orders of magnitude. Therefore, the CMC values for all [Cn]­[MCPA] studied here are expected to lie within the range of 0.1–10 mM. Under these conditions, only monomeric [Cn]­[MCPA] species are expected to be present in the subphase, unless the interface is fully occupied by lipids.

The π-A isotherms of DPPC, CHOL, DMPE, and ERG, recorded in the presence of 0.07–0.69 μmol/L of [C12]­[MCPA] in the subphase, are shown in Figure 4a–d. A consistent trend across all panels is an expanding effect caused by [C12]­[MCPA], intensifying with the increasing concentration of the salt. For the DPPC monolayer in the presence of 0.07 and 0.14 μmol/L of [C12]­[MCPA], an expanding effect is seen up to π = 20 mN/m. At higher concentrations of HIL, the expanding effect is visible up to ∼40 mN/m. This means that compression leads to expelling the soluble molecules of herbicides from the interface, and this is why the area occupied per molecule comes back to the value for pure DPPC. In the presence of [C12]­[MCPA], a typical plateau shown in the DPPC isotherm at 52–70Å^2^/molec. is not visible in the presence of herbicides, which means the monolayer is affected mostly by [C12]­[MCPA] at low packing density of the film. This is in contrast to CHOL, which exhibits strong expansion in the whole range of surface pressures. π_coll._ of the CHOL monolayer is slightly elevated in the presence of [C12]­[MCPA], indicating that the film can withstand higher lateral pressures before it transitions to three-dimensional structures at the interface. In contrast, ERG shows a strong π_coll._ reduction of up to 16 mN/m under the same conditions, demonstrating that [C12]­[MCPA] destabilizes the ERG monolayer while exerting a stabilizing effect on CHOL. This contrasting behavior likely arises from subtle yet critical differences in the chemical structures of both sterols. Cholesterol’s planar and compact structure facilitates tight lipid packing and enhances membrane stability, whereas ergosterol’s more rigid but less planar structure results in less ordered monolayers, which are inherently more vulnerable to destabilization by [C12]­[MCPA]. The expanding effect in the ERG monolayer is less pronounced than for the CHOL film, and for the largest concentrations of herbicides, the changes of slope affect the molecular area intensively. In the case of the DMPE monolayer, the expansion is strong in the whole range of surface pressures only for the highest concentrations of herbicides. Furthermore, in most cases, the DPPC monolayer collapses at higher surface pressures in the presence of the herbicide than pure phospholipid.

**Figure 4:**
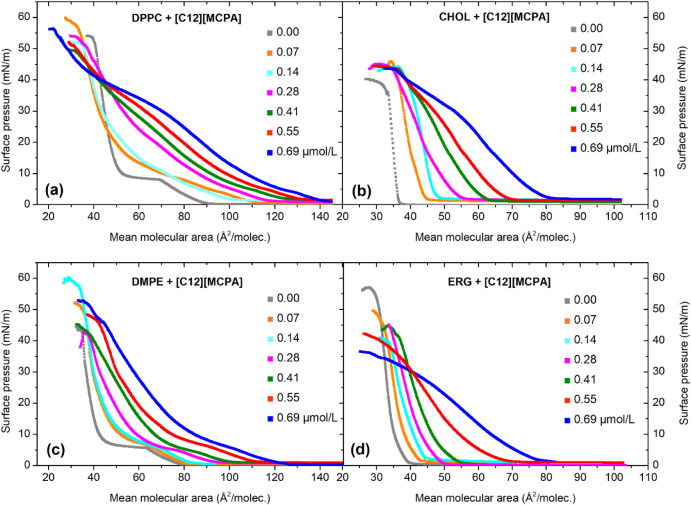
Surface pressure–area isotherms for DPPC (a), CHOL (b), DMPE (c), and ERG (d) recorded at various concentrations of [C12]­[MCPA] in the subphase.

Since the addition of HIL to the subphase results in a clear expansion of the monolayer area, we decided to further quantify this phenomenon under biologically relevant conditions. Therefore, we analyzed the mean molecular area values at a fixed surface pressure of π = 30 mN/m for various concentrations of [C12]­[MCPA]. As illustrated in Figure 5, the DPPC monolayer exhibits the most remarkable change in A_30_ with increasing concentration of HIL, indicating a strong interaction and incorporation of [C12]­[MCPA] into the phospholipid film. In contrast, the concentration-dependent variation of A_30_ is considerably weaker for CHOL and DMPE and remains similar for both of these lipids. The ERG monolayer shows the weakest dependence on concentration changes.

**Figure 5:**
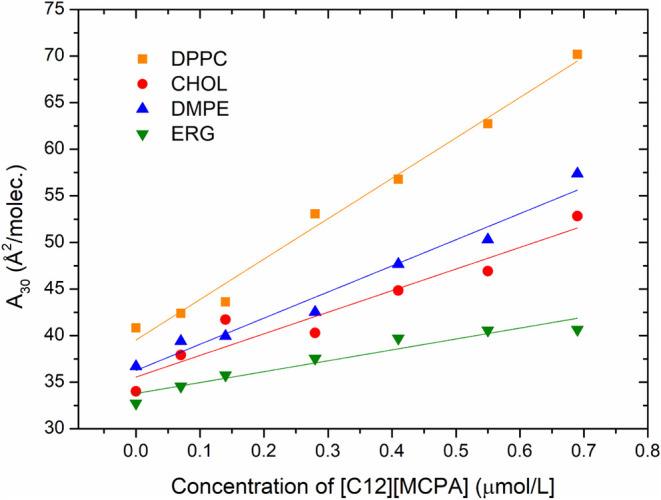
Mean molecular areas determined at π = 30 mN/m for DPPC, CHOL, DMPE, and ERG monolayers at various concentrations of [C12]­[MCPA] in the subphase.

The results presented in Figure 5 provide a useful framework for predicting the dose-dependent effects of [C12]­[MCPA] on various membrane types. The data indicate that increasing the concentration of [C12]­[MCPA] amplifies its adverse effect on DPPC-rich membranes while exerting only a minor influence on the ergosterol film.

Membrane fluidity changes induced by increasing the [C12]­[MCPA] concentration were further analyzed by comparing the compression modulus curves. As shown in Figure 6, the addition of [C12]­[MCPA] leads to noticeable fluidization of most monolayers, indicating that the films become less ordered in the presence of the HIL. Among all systems, the compression modulus of the ERG monolayer demonstrates the strongest sensitivity to changes in [C12]­[MCPA] concentration, an observation that contrasts with the relatively modest variation in the A_30_ value shown in Figure 5. This discrepancy suggests that the increasing HIL concentration affects molecular ordering and packing dynamics rather than causing additional film expansion of the ERG film. The less pronounced changes in fluidity with varying salt concentrations are observed for DMPE and CHOL. For DPPC, the reduction of fluidity in the presence of [C12]­[MCPA] is limited.

**Figure 6:**
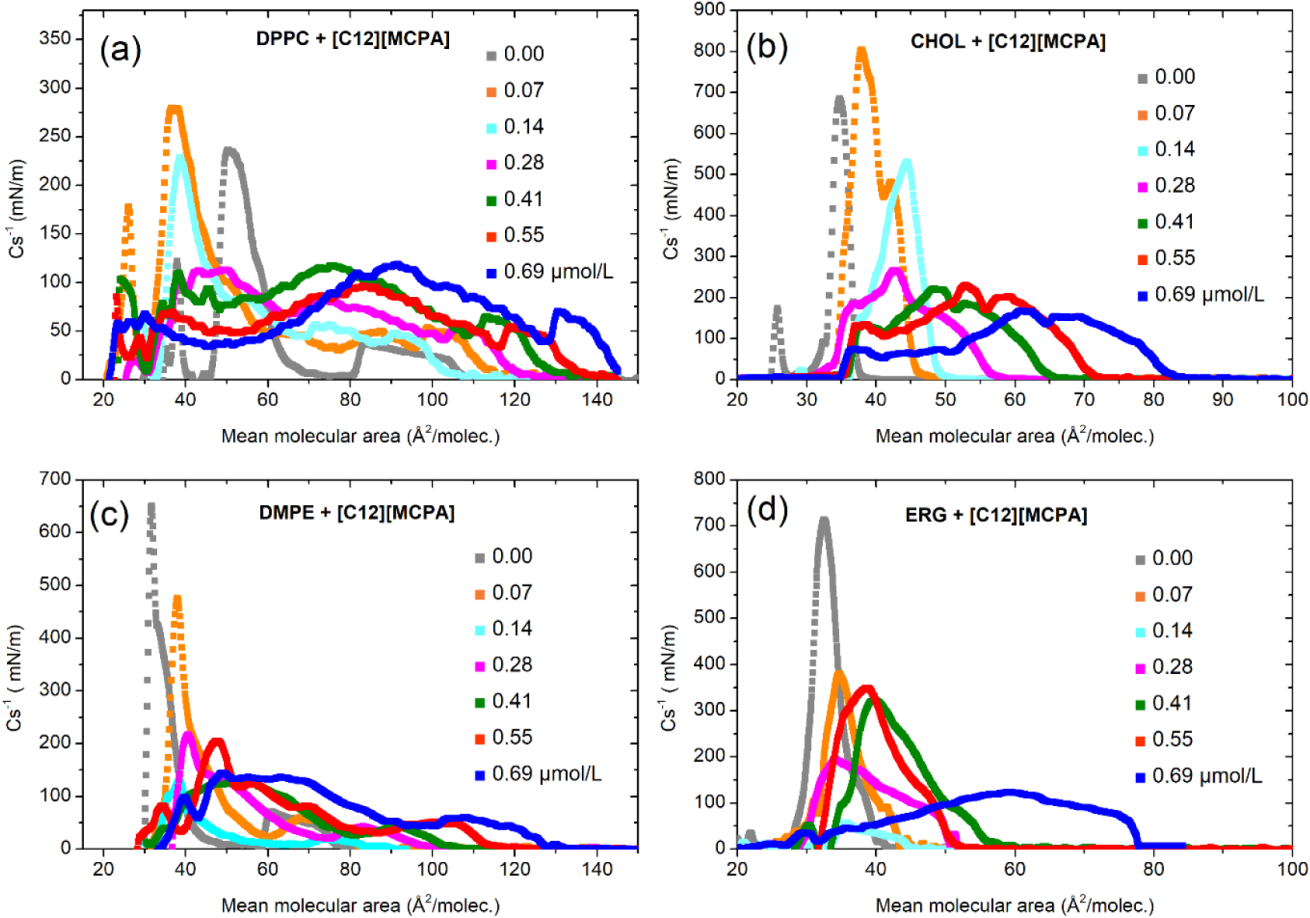
Compression modulus plotted against the mean molecular area for DPPC (a), CHOL (b), DMPE (c), and ERG (d) monolayers in the presence of [C12]­[MCPA] salts in the subphase. The concentration of the salt in the subphase varies from 0.14 to 0.69 μmol/L. The calculations were performed by using the π–A isotherm data shown in Figure 4.

When approaching the membrane from the aqueous phase, the herbicide acts as a soluble amphiphile that must partition into the condensed lipid layer to exert its perturbing effect. To mimic this initial contact, a penetration experiment was performed by injecting [C12]­[MCPA] solution beneath precompressed lipid monolayers. Under these conditions, the monolayer is less hydrated, and its permeability is largely determined by the molecular packing density. The dynamic response of the monolayer to HIL injection was evaluated at two concentrations, 0.69 and 2.06 μmol/L. The resulting relative area changes over time are shown in Figure 7 along with corresponding curves for pure lipids compressed to the same target surface pressure. Pure lipid monolayers exhibit a small to moderate decrease in the relative area, with the most pronounced instability observed for DMPE and ERG, indicating weaker lateral cohesion within these films. In contrast, the A/A_0_ curves for pure DPPC and CHOL films remain relatively stable throughout the measurement period.

**Figure 7:**
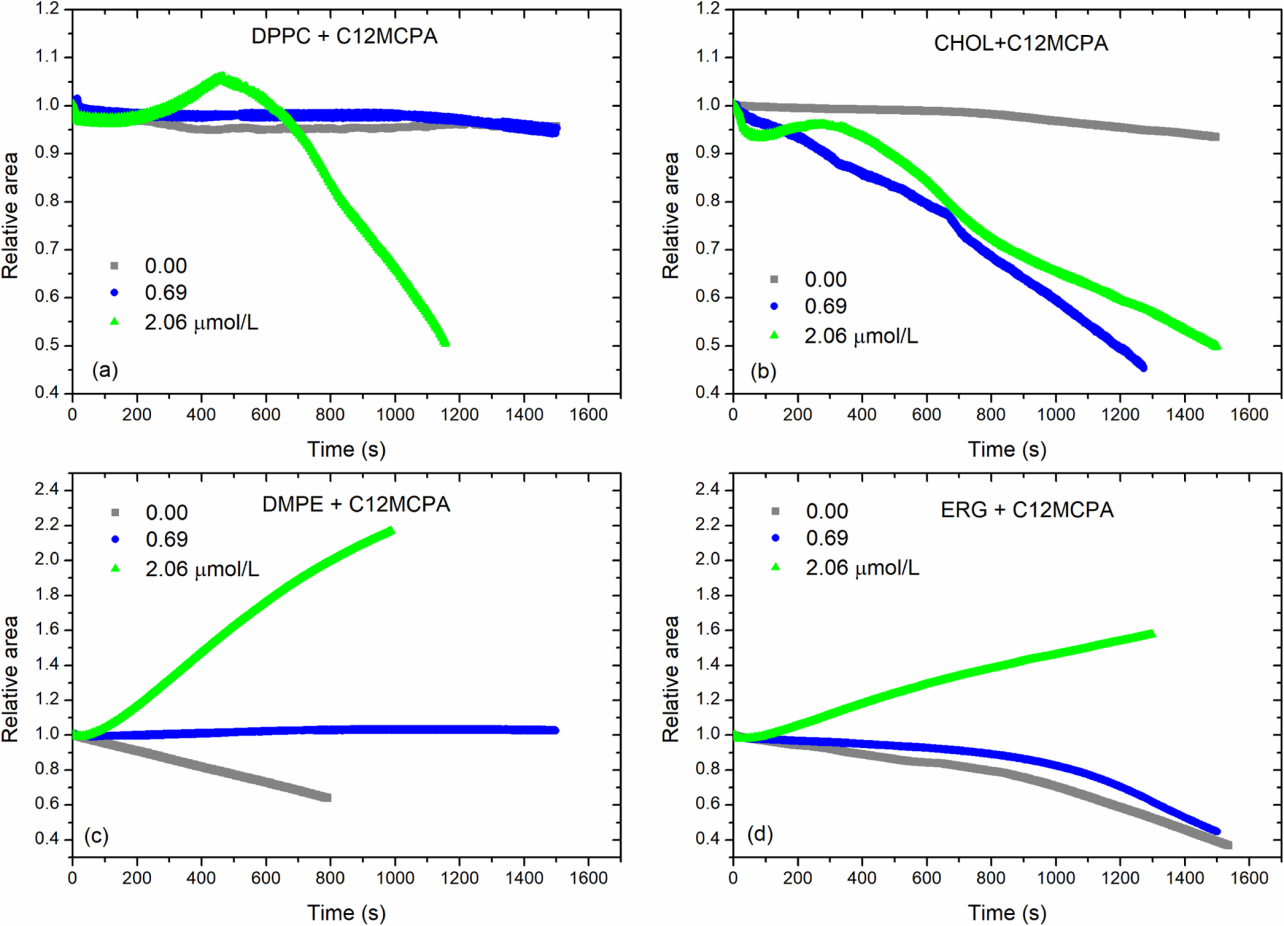
Relative area changes over time for DPPC (a), CHOL (b), DMPE (c), and ERG (d) monolayers compressed to π = 30 mN/m (gray lines) together with the curves recorded after injection of [C12]­[MCPA] solution to the subphase. The final concentrations of [C12]­[MCPA] in the subphase were 2.06 μmol/L (green lines) and 0.69 μmol/L (blue lines). The injection was performed at *t* = 0 under continuous stirring of the subphase.

The behavior illustrated in Figure 7 following [C12]­[MCPA] injection can be interpreted in terms of the competition between lipid and amphiphilic herbicide molecules for interfacial area and the reversibility of adsorption–desorption processes governed by geometric and packing constraints. At higher herbicide concentration (2.06 μmol/L), DMPE and ERG monolayers exhibit the strongest expansion, with A/A_0_ values reaching 2.2 and 1.4, respectively, at *t* ∼ 1000 s. Conversely, the DPPC film shows only a transient increase of the relative area followed by a decrease below unity, indicating weak tendency of the herbicide to adsorb between the lipid molecules at the interface. In that case, the interaction of the herbicide with the monolayer even accelerates the loss of the lipid species from the interface. A similar observation can be made for the CHOL monolayer.

At the lower concentration of [C12]­[MCPA], the expansion of the DMPE + [C12]­[MCPA] film is less pronounced, yet remains significantly greater than that of pure DMPE. The plateau near 1000 s indicates the establishment of a dynamic equilibrium between adsorption and desorption of the molecules at the interface. For ERG + [C12]­[MCPA], the concentration of 0.69 μmol/L is insufficient to induce measurable expansion; instead, a gradual decrease in A/A_0_ followed by the inflection point is observed, possibly reflecting film bending and eventual collapse.

Interestingly, for the CHOL monolayer, both concentrations of HIL lead to a large decrease in the relative area, suggesting extensive loss of material from the interface. This behavior is likely associated with the formation of three-dimensional lipid-surfactant aggregates, which reduce the mean molecular area. Once the available free area is saturated, further insertion of [C12]­[MCPA] molecules between closely packed sterol molecules becomes unfavorable, promoting the nucleation of 3D aggregates instead of additional interfacial adsorption.

Although DPPC forms a more fluid and hydrated film than DMPE, enhanced penetration of the herbicide is observed for DMPE, representing the lipids abundant in plant and bacterial plasma membranes. The pronounced expansion of the tightly packed DMPE film upon exposure to [C12]­[MCPA] likely arises from flexibility and optimal hydrophobic matching between their chains (C14 and C12). In the case of DPPC, the initial rise of the relative area at *c* = 2.06 μmol/L followed by the sharp decrease is in agreement with the observations made in the isotherm experiment (Figure 1a). The herbicide molecules adsorb at the interface, but apparently, due to competition between the soluble amphiphile and an insoluble lipid, the molecules of [C12]­[MCPA] desorb to the subphase.

The rigid, bulky structures of sterols are potentially less susceptible to perturbation in the condensed state than those of phospholipids. However, the different responses of CHOL and ERG monolayers to the same amounts of [C12]­[MCPA] suggest there is a more complex mechanism governing the insertion of the herbicide to the lipid film. A distinct area expansion observed for ERG + [C12]­[MCPA] at higher concentration (2.06 μmol/L) highlights the concentration-dependent nature of the herbicide-ergosterol interaction. However, a subtle difference in the bulkiness of CHOL and ERG resulting from various unsaturation degrees and additional methyl groups in ERG molecules can facilitate the adsorption of [C12]­[MCPA] within the film.

Overall, the varying effects of [Cn]­[MCPA] salts on lipid monolayers indicate that their biological activity might be related to the membrane composition. Given the critical role of membrane fluidity in processes such as nutrient transport, signal transduction, and cellular homeostasis, perturbations induced by these herbicidal ionic liquids could have a significant impact on both target and nontarget organisms. The largest differences in membrane response could be anticipated for herbicides with the shortest (C8) and the longest alkyl chains (C16, C18), whereas the derivatives with C12 and C14 chains appear to be the most potent membrane perturbants, albeit less selective. Penetration experiments demonstrated that condensed membranes rich in DMPE and ERG are the most sensitive to the presence of [C12]­[MCPA], although this effect is dose-dependent. Notably, distinct membrane behavior in the presence of herbicides was observed for CHOL and ERG, suggesting that [Cn]­[MCPA] may exert different effects on fungal and animal cell membranes. Together, these insights provide a foundation for predicting molecular interactions and toxicity profiles in biological membranes, thereby facilitating the rational design of herbicides with minimized off-target effects.

## Conclusions

This study elucidates the influence of the alkyl chain length and concentration of herbicidal ionic liquids [Cn]­[MCPA] on the structural and mechanical properties of model lipid monolayers composed of phospholipids (DPPC, DMPE) and sterols (CHOL, ERG). The effects of [Cn]­[MCPA] salts on lipid monolayers are strongly dependent on the alkyl chain length and concentration of the herbicide, with dodecyl and tetradecyl derivatives exerting the largest fluidizing and expanding effects on all films.

The dose dependence of these effects is evident, as increasing concentrations of [C12]­[MCPA] result in greater monolayer expansion, enhanced fluidity, and distinct destabilization behavior depending on the lipid type. Among the tested systems, the DPPC monolayer was the most responsive to varying concentrations of [C12]­[MCPA].

Penetration experiments, simulating the dynamic contact of the herbicide with a biological membrane, revealed a particularly high ability of [C12]­[MCPA] to insert the condensed DMPE and ERG monolayers, with the magnitude of this effect intensifying at higher herbicide concentrations. These findings further demonstrate that membrane composition and molecular packing strongly dictate responsiveness: phospholipid monolayers accommodate amphiphile insertion more readily, whereas sterol-rich films resist adsorption yet exhibit increased susceptibility to aggregate formation and interfacial instability at lower herbicide doses. The results also suggest a potential mechanism of monolayer collapse through the formation of lipid-herbicide assemblies, observed for both sterol monolayers.

Moreover, our study suggests that a biological effect of the herbicide with the MCPA ion can be modulated by reasonable selection of the alkyl chain length and concentration of HIL.

Overall, our findings indicate that the biological activity associated with membrane perturbation by herbicidal ionic liquids can be partially predicted by using simple in vitro techniques, such as the Langmuir monolayer approach. This approach enables a detailed examination of interactions between individual lipids and soluble amphiphiles, thereby providing molecular-level insight into physicochemical processes occurring at biological interfaces. In addition, the method offers distinct advantages, including low cost, rapid analysis, and precise control of experimental conditions, making it a valuable tool for predicting membrane interactions of amphiphilic compounds and assessing their potential biological or environmental impact.

## Supplementary Material



## Notes

π–A isotherms recorded for pure [C12]­[MCPA] (PDF)
