# Supplementary material for: Lipid Matters: How Herbicidal Ionic Liquids Engage with Membranes
Source: J Phys Chem B. 2026 Jan 21;130(5):1704–15. doi: 10.1021/acs.jpcb.5c07127 (PMC13296725; doi:10.1021/acs.jpcb.5c07127)
Supplement: Supplementary file 1 [file jp5c07127_si_001.pdf]

# Lipid Matters: How Herbicidal Ionic Liquids Engage with Membranes

Aleksandra Bagińska, Anna Syguda, Katarzyna Dopierała*

*Institute of Chemical Technology and Engineering, Poznan University of Technology, Berdychowo 4, 60−965 Poznań, Poland*

*corresponding author, E−mail: katarzyna.dopierala@put.poznan.pl

**Supporting Information**

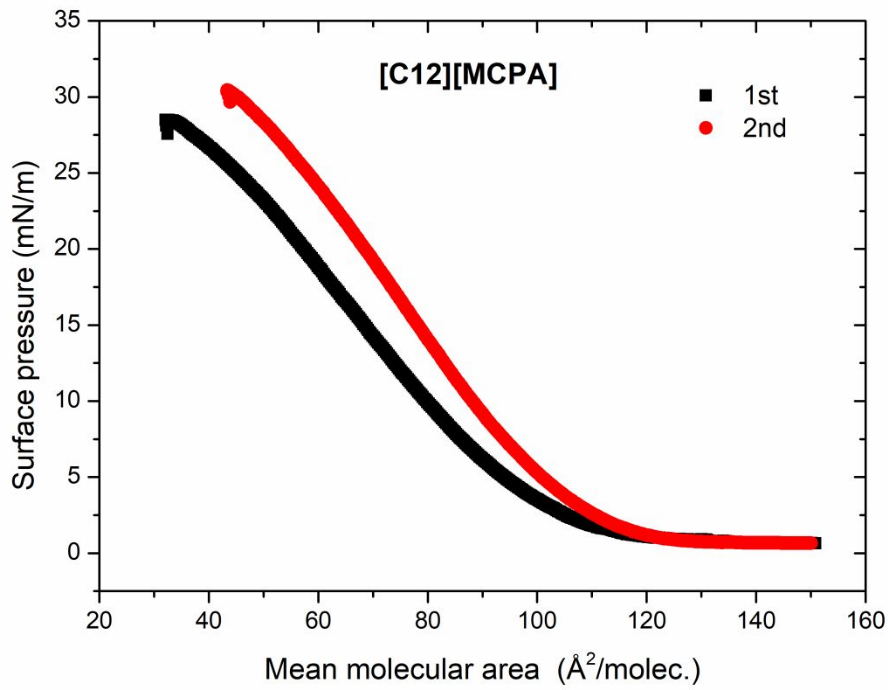


**Figure S1.** The $\pi$-A isotherms for C12MCPA spread at the air/water interface from chloroform solution (1mg/mL). The 1$^{st}$ and 2$^{nd}$ curve represent two independent experiments.

The molecular areas obtained by extrapolating the slope of the $\pi$-A isotherms to zero-surface pressure pressure ($A_0$) are 112 and 116 Å$^{2/}$molec. for the 1$^{st}$ and 2$^{nd}$ run, respectively.

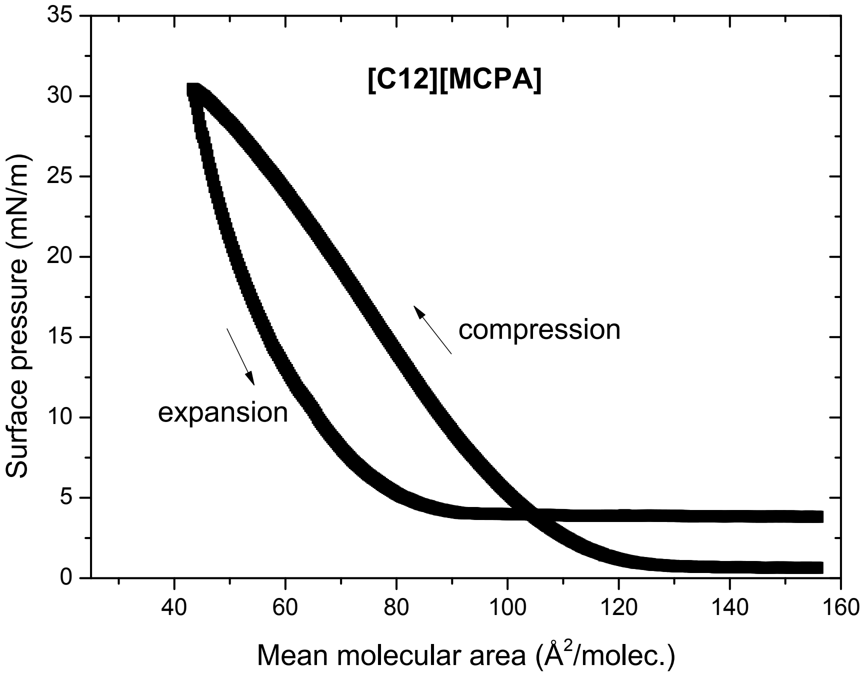


**Figure S2.** The compression-expansion curve for [C12][MCPA] recorded after spreading from chloroform solution.

A pronounced hysteresis observed during compression-expansion cycles might be associated with gradual desorption of the molecules from the interface or irreversible aggregation

Taken together, the results shown in Figures S1-S2, we concluded that [C12][MCPA] alone does not form a true, stable Langmuir monolayer alone despite exhibiting significant surface activity.
